# PROBLEMS AND PROSPECTS FOR CURRENT POLICIES TO EXTEND WORKING LIVES

**DOI:** 10.1093/geroni/igz038.3024

**Published:** 2019-11-08

**Authors:** Debra A Street, Debra A Street

**Affiliations:** State University of New York at Buffalo, Buffalo, New York, United States

## Abstract

Current policy debates fluctuate between “extending working life” and “delaying retirement”—assuming policies that reflect different conceptual approaches are identical. This presentation uses a different analytic strategy, conceptualizing later life work policies as representing distinctive approaches to consideration of work conditions/income security for older workers. Using data from over 30 countries, I discuss main trends in extended working life policies (mainly in the EU) and the gender and health implications for current and future workers. We find that policies committed to “extending working life”—supporting adequate/meaningful employment for later life work—are enacted rarely, but with potentially positive effects for the health and wellbeing of older workers of either gender. However, “delaying retirement” policies, which dominate the political landscapes of most of the country-specific policies we consider, reproduce or exacerbate gender inequalities and health risks for vulnerable older workers.

